# Plasmonic and Conductive Structures of TCO Films with Embedded Cu Nanoparticles

**DOI:** 10.3390/ijms231911886

**Published:** 2022-10-06

**Authors:** Stefano Boscarino, Valentina Iacono, Andrea Lo Mastro, Fiorella Tringali, Antonio Terrasi, Maria Grazia Grimaldi, Francesco Ruffino

**Affiliations:** 1 Dipartimento di Fisica e Astronomia “Ettore Majorana”, Università di Catania, Via S. Sofia 64, 95123 Catania, Italy; 2 CNR-IMM, Via S. Sofia 64, 95123 Catania, Italy; 3 Research Unit of the University of Catania, National Interuniversity Consortium of Materials Science and Technology (INSTM-UdR of Catania), Viale Andrea Doria 8 and Via S. Sofia 64, 95125 Catania, Italy

**Keywords:** copper, zinc oxide, AZO, indium oxide, IZrO, nanostructures, laser ablation, dewetting, plasmonic, solar cells

## Abstract

Cu nanoparticles were produced by using solid-state dewetting (dry) of a 1.3 nm Cu layer or laser ablation of a Cu solid target (wet) in acetone and methanol. The morphology and chemical composition of the nanoparticles were investigated as a function of the synthesis methods and their key parameters of the annealing temperature (200–500 °C) and the liquid environment during the ablation. Cu nanoparticles were then embedded in transparent conductive oxide (TCO) films as aluminum-doped zinc oxide (AZO) or zirconium-doped indium oxide (IZrO); the TCO_bott_/Cu nanoparticle/TCO_top_ structures were synthesized with all combinations of AZO and IZrO as the top and bottom layers. The goal was to achieve a plasmonic and conductive structure for photovoltaic applications via a comparison of the involved methods and all fabricated structures. In particular, solid-state dewetting produced faceted or spherical (depending on the annealing temperature) nanoparticles with an average size below 150 nm while laser ablation produced spherical nanoparticles below 250 nm. Dry and wet plasmonic conductive structures as a function of the TCOs employed and the temperature of annealing could reach a sheet resistance of 86 Ω/sq. The energy band-gap E_gap_, absorbance, transmittance, and reflectance of the plasmonic conductive structures were investigated in the UV–vis–NIR range. They showed a dependence on the sequence of the top and bottom TCO, with best transmittances of 89.4% for the dry plasmonic conductive structure and 84.7% for the wet plasmonic conductive structure. The latter showed a higher diffused transmittance of between 10–20% in the visible range.

## 1. Introduction

In the transition toward clean and renewable energy in recent years, photovoltaic (PV) technology has become a strategic field that, thanks to its low costs (over the last decade the cost of solar power has decreased by 82%), ease of manufacturing, and other benefits, can provide renewable, sustainable, and environmental friendly energy [1,2]. A crucial factor that determines the performance of solar cells (not including their generation) with their absorbent characteristic layer and technology is the efficiency of the light absorption (light harvesting) needed to generate electron–hole pairs. A conventional method implemented in solar cells to increase the path length within the absorbent layer is a textured surface such as a random pyramid or inverted pyramid, which produces the scattering of sunlight over a broad angular range and consequently increased the path length, promoting an enhancement of the light-harvesting performance in solar cells [3,4,5].

In order to overcome problems related to the use of a textured surface and to produce more efficient light scattering, metal nanostructures, with their well-known optical property of surface plasmon resonance (SPR), have been demonstrated to be one of the best solutions to enhancing and scattering the incident sunlight [6,7,8,9,10,11]. In details, SPR becomes a localized surface plasmon resonance (LSPR) due to the reduced dimensions of a nanostructure, which localize oscillations of electrons. Many factors influence the intensity and wavelength of LSPR, such as the type of metal used, the size and shape of the nanostructure, the dielectric properties of the nanostructure, and the dielectric constant of the surrounding medium.

Copper (Cu) has become a great alternative to the most used, studied, and high-priced noble metals such as gold (Au) and silver (Ag, thanks to its low-cost [12,13,14], earth-abundant nature, and other characteristics that can prop up its use on a large scale in the manufacturing of PV cells. Moreover, Cu is characterized by an LSPR in the 500–800 nm range, although it often shows a broad and weak LSPR at ~590 nm. In recent years, in order to achieve a narrow and intense peak and also to prevent the oxidation process, strong efforts have been made and different technological solutions have been proposed and carried out. To mention a few: different shapes (Cu nanotriangle, Cu half-shell, and cubic shape) [12,13,14,15,16]; chemical and physical methods such as solid-state dewetting (SSD), nanosphere lithography, e-beam lithography, focused-ion-beam machining, chemical reduction, electrochemical synthesis, and laser ablation of solid target in solvents (LAL) such as ethanol, ethylene glycol, glycol, acetone, and methanol; and Cu nanostructures embedded into different matrices [12,15,16,17,18,19,20,21,22].

In this work, we focused on solid-state dewetting (dry method) and laser ablation of a solid target in a solvent (wet method) to produce Cu nanostructures that in turn were embedded in thin layers of transparent and conductive oxides (TCOs) of AZO (aluminum-doped zinc oxide), IZrO (Zirconium-doped Indium oxide), and a mix of both. We chose these TCOs due to their compatibility with solar cell manufacturing and, more importantly, their indium-free (or very low indium consumption) transparent electrodes or buffer layer [23,24,25]. Briefly, the SSD method is based on a thermally induced modification of morphology well below the melting point of a metal thin film into an array of droplets or nanostructures/nanoparticles. The key factors that determine the resulting nanostructures’ morphology are the thickness of the starting metal film, the temperature of the process, and the interaction between the substrate and the metal film [26]. In contrast, in the LAL process, a laser beam is focused on the surface of a solid target and immersed in a solvent that absorbs the laser radiation, which under suitable conditions leads to the nucleation and growth of metal particles that are released into the surrounding solvent, hence forming a nanoparticle colloidal solution [27]. The key factors that determine the resulting nanostructures’ morphology are, in this case, the laser parameters (laser wavelength and fluence, pulse duration, and repetition rate) and the liquid environment in which the ablation is carried out.

In this work, Cu nanoparticles were produced by means of dry SSD of a 1.3 nm Cu layer and wet LAL of a Cu target using a 1064 nm nanosecond-pulsed laser in acetone and methanol. The morphology of the Cu nanoparticles (size and shape) was examined as a function of the synthesis methods and their key parameters: in the SSD, the morphology was studied as a function of the annealing temperature of 200 to 500°C in N_2_ for 1 h; while in the LAL, the morphology was studied as a function of the employed liquid environment. Moreover, the chemical composition of the Cu nanoparticles and TCOs layers was studied by using energy-dispersive X-ray analysis. The produced Cu nanostructures were then embedded in TCOs as an AZO layer, an IZrO layer, or a mix of both TCO. The TCO_bott_/Cu nanoparticle/TCO_top_ structures were synthesized using all combinations of AZO and IZrO as the top and bottom layers. The goal of this research was to achieve a plasmonic and conductive structure (PCS) for photovoltaic applications via a comparison of the involved methods and all created structures.

The differences observed between the wet and dry processes were in the shape and size of the Cu nanoparticles: the SSD produced faceted or quasispherical nanoparticles with an average size below 150 nm, while the LAL produced spherical nanoparticles smaller than 50 nm in acetone and larger but smaller than 250 nm for methanol. Dry and wet PCSs as a function of the TCOs employed and the temperature of annealing could achieve a sheet resistance of 86 Ω/sq in the best conditions for the dry PCS and ~250 Ω/sq at room temperature for the wet and dry PCSs. Optical properties such as the energy band gap, direct transmittance, absorbance, and specular or total reflectance were investigated in the 250–1100 nm wavelength range. They showed a strong dependence on the sequence of the top and bottom TCOs and type (wet or dry) of Cu nanoparticles embedded in them. The values of the Egap in the PCSs strongly depended on the type of top and bottom TCOs employed: the TCO with the lowest Egap imposed the Egap to the whole PCS. The best dry PCS exhibited a maximum transmittance of 89.4% at 754 nm with a mean value of transmittance in the visible range of 78.7% and visible–NIR range of 80.8% compared to the best wet PCS (in acetone), which had a maximum of 84.7% at 737 nm and a mean value of transmittance of 74.7% in the visible range and 76.9% in the visible–NIR range. Moreover, the best wet PCS showed the highest diffused transmittance of between to 10–20% in the visible range as compared to the best dry PCS.

## 2. Results and Discussion

### 2.1. Chemical Composition and Morphology Analysis

**Figure 1:**
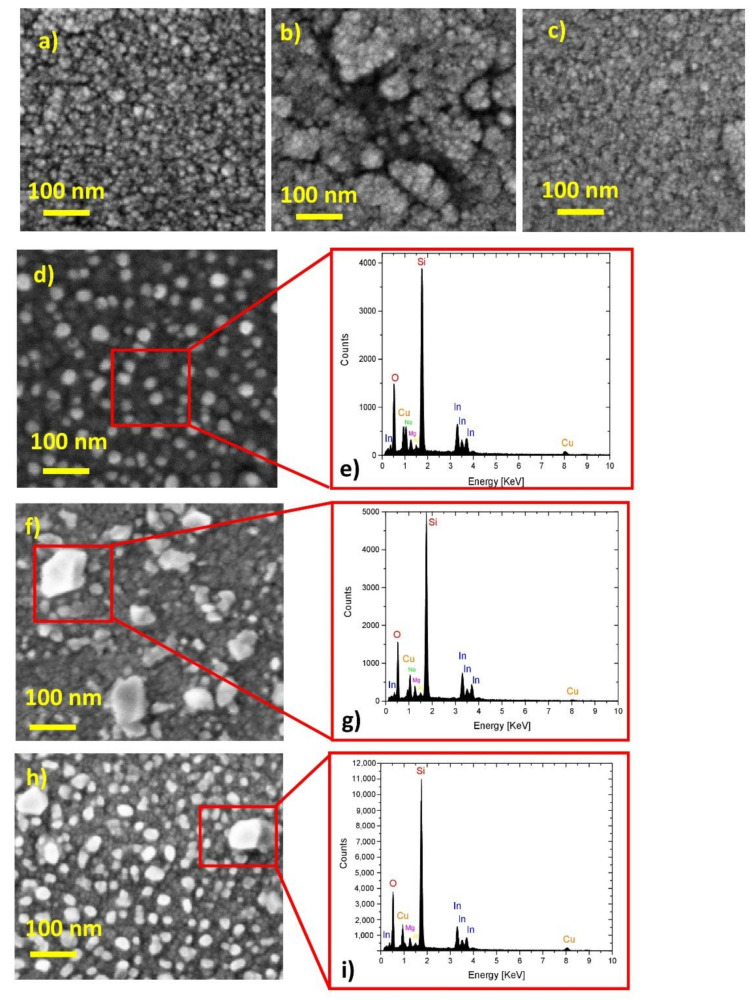
SEM images of (**a**) as-deposited IZrO botttom layer surface; (**b**) the surface of as-deposited Cu on IZrO bottom layer; (**c**) glass substrate/IZrO bottom/Cu stack after a thermal annealing at 200 °C; (**d**) glass substrate/IZrO bottom/Cu stack after a thermal annealing at 300 °C; (**e**) EDX spectrum of the sample shown in (**d**) acquired over the Cu nanostructures on the position indicated by the red square; (**f**) glass substrate/IZrO bottom/Cu stack after a thermal annealing at 400 °C; (**g**) EDX spectrum of the sample shown in (**f**) acquired over the Cu nanostructures on the position indicated by the red square; (**h**) glass substrate/IZrO bottom/Cu stack after a thermal annealing at 500 °C; (**i**) EDX spectrum of the sample shown in (**h**) acquired over the Cu nanostructures on the position indicated by the red square.

The chemical composition, morphology, and size of the Cu nanoparticles (Figure 1) produced on the AZO or IZrO bottom layers by the dewetting process of a deposited film as a function of the thermal treatment temperature that induced the dewetting were investigated using SEM-EDX and RBS.

First of all, the RBS analysis (Figure S1) of our samples showed a stoichiometry compatible with the known composition of the AZO and IZrO targets; moreover, it confirmed the following: the 0.6 at.% Zr doping in In_2_O_3_; the thickness of our deposited films for AZO, IZrO, and Cu; and no oxidation (no presence of oxygen) of Cu (as deposited) film sputtered on TCO.

Figure 1a shows the surface of the IZrO bottom layer; as expected for this type of deposition, we observed a granular structure related to an amorphous nature of the TCO [25,28]. Cu was sputtered uniformly and conformally on the surface of the IZrO bottom layer (Figure 1b). The early stage of growth of the thin film was highlighted by the nucleation or phenomena of aggregation, as well as the underlying IZrO granular structure visible from the breakage in the copper film.

After a thermal annealing at 200 °C (Figure 1c), no significant modification in the structure of the Cu film was observed. At 300 °C, the solid-state dewetting process (Figure 1d) was achieved and resulted in the formation of Cu nanoparticles with quasispherical and spherical shapes.

When increasing the temperature up to 400 and 500 °C (Figure 1f,h, respectively), we observed an increasing material agglomeration with the formation of larger faceted Cu nanoparticles with an average diameter of ~120 nm. These results confirmed that the solid-state dewetting process resulted in the formation of a particle distribution dominated at temperatures higher than 300 °C by large-sized faceted nanoparticles.

After the deposition of the IZrO or AZO top layer, SEM images (not reported here) showed our nanoparticles were covered by a compliant and continuous TCO layer with an increased average size. Moreover, the Cu thin film showed the same evolution as a function of the annealing temperature when it was deposited on the AZO bottom layer.

In addition to the morphological SEM analysis, we also carried out a chemical compositional analysis of the dewetted Cu nanoparticles by acquiring the EDX spectrum of the Cu nanoparticles supported on the IZrO layer for each annealing temperature (Figure 1e,g,i). In all the EDX spectra, we observed the following characteristic features: Cu lines related to Cu nanoparticles; In lines related to the IZrO bottom layer; and Si and O lines related to the glass substrate (SiO_2_) and SiO_2_ or IZrO layer, respectively (the RBS analysis reported in Supplementary Materials Figure S1 supported the absence of oxygen in the Cu thin film); and Mg, Al, and Na lines attributed to contamination in the glass substrate.

**Figure 2:**
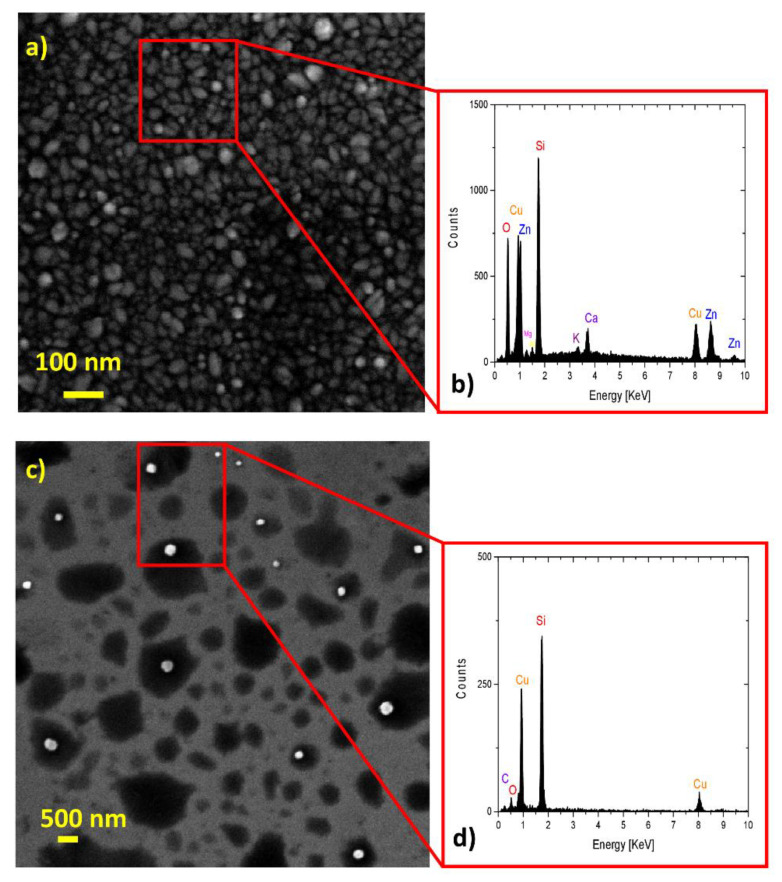
(**a**) SEM image of Cu nanoparticles produced in acetone on AZO bottom layer; (**b**) EDX spectrum of the sample shown in (**a**) acquired over the Cu nanostructures on the position indicated by the red square; (**c**) SEM image of Cu nanoparticles produced in methanol deposited on Si layer; (**d**) EDX spectrum of the sample shown in (**c**) acquired over the Cu nanostructures on the position indicated by the red square.

Figure 2 reports on the SEM-EDX analysis of the Cu nanoparticles obtained via laser ablation in acetone and methanol at a power density of 5 J cm^−2^ per pulse and for an 8 min ablation time and then deposited on the AZO or IZrO bottom layer.

In detail, Figure 2a shows the SEM micrograph for the Cu nanoparticles produced via laser ablation in acetone deposited on top of the AZO bottom layer. The surface of the AZO bottom layer with a symmetric granular structure related to a highly (002) textured columnar polycrystalline films with a wurtzite structure [24,25] is clearly visible. In addition, Figure 2c shows the Cu nanoparticles produced via laser ablation in methanol and deposited on top of the Si layer. As can be seen, laser ablation in methanol and acetone produced spherical nanoparticles with a larger average diameter in methanol than in acetone. In particular, when produced in acetone, the average diameter of particles was ~ 33 nm, while it was ~230 nm when produced in methanol. The results for the Cu NPs in acetone were in agreement with the growth mechanism, which, as a function of dipole moment and the higher moment of acetone, produced smaller nanoparticles [29,30].

In addition, a chemical compositional analysis of the laser-ablated Cu nanoparticles was performed by acquiring the EDX spectrum of the Cu nanoparticles deposited on the AZO layer (for acetone) and on Si (for methanol) (Figure 2b,d).

In the EDX spectra acquired for the Cu nanoparticles (produced in acetone) deposited on the AZO layer, we observed the following characteristic features: Cu lines related to Cu nanostructures; Zn lines related to the AZO bottom layer; Si and O lines related to the glass substrate (SiO_2_) and SiO_2_ or AZO layer, respectively; and Mg, Al, Ca, and K lines attributed to contamination in the glass substrate.

For the EDX spectra acquired for the Cu nanoparticles (produced in methanol) deposited on an Si layer, we observed the following characteristic features: Cu lines related to Cu nanostructures, an Si line related to the Si substrate, an O line related to native silicon oxide on the silicon (Si) surface, and C lines related to the solvent.

### 2.2. UV–Vis–NIR Optical Properties

**Figure 3:**
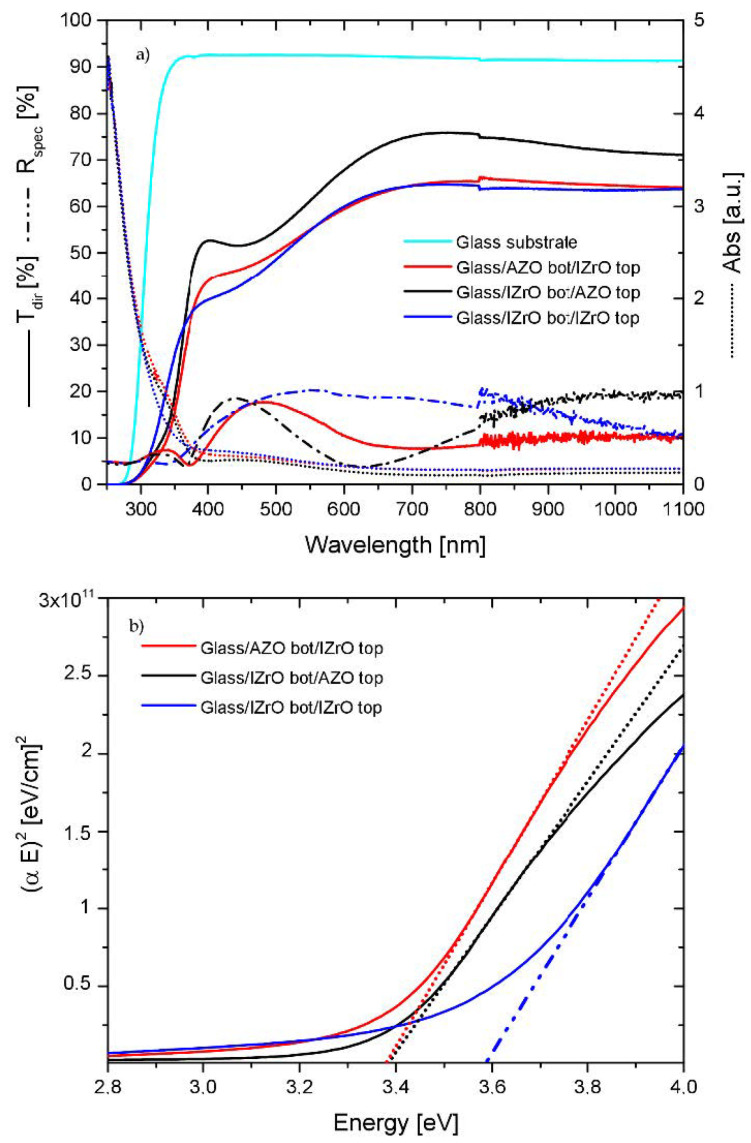
(**a**) Transmittance, reflectance, and absorbance curves of Glass/AZO_bott_/IZrO_top_, Glass/IZrO_bott_/AZO_top_, and Glass/IZrO_bott_/IZrO_top_ double stack. The transmittance of the bare glass substrate is reported as a reference. (**b**) Tauc plots and calculated linear fit of the samples reported in (**a**).

Figure 3a shows the optical direct transmittance, specular reflectance, and absorbance spectra of the Glass/AZO_bott_/IZrO_top_, Glass/IZrO_bott_/AZO_top_, and Glass/IZrO_bott_/IZrO_top_ double stack; while Figure 3b shows their optical band gaps evaluated by using Tauc’s equation for direct transitions. Both the IZrO and AZO TCOs had wide direct band gaps.

Tauc’s law for direct transitions is based on the assumption that the energy-dependent absorption coefficient *α* can be expressed by the following equation [25,31]:

(1) $${\left(E\alpha \right)}^{2}=B\left(E-E_{gap}\right)$$

where *E* is the photon energy, *α* is the absorption coefficient, and *B* is the Tauc coefficient. In this way, we obtained the E_gap_ from the energy axis intercept of the line fitted on the linear portion of the Tauc plots. In our samples, *α* was calculated by using the following equation [32]:

(2) $$\alpha_{PCS}=\frac{1}{d_{PCS}}In\frac{T_{Glass\;}\left(1-R_{PCS}\right)}{T_{PCS}}$$

where d_PCS_, T_PCS_, and R_PCS_ are the thickness, direct transmittance, and specular reflectance of the PCS structure, respectively; while T_Glass_ is the direct transmittance of the bare glass substrate.

Moreover, the transmittance of glass substrate was reported and the data included the substrate contribution. These data and optical behaviors of the double stack were essential as references in order to compare and understand the optical properties of the dry and wet PCS.

As can be seen, depending on the sequence of top and bottom TCO layers in our double stack samples, we changed the transmittance of samples because we were changing the reflectance. This behavior could be ascribed to the differences in the refractive indices of the IZrO and AZO thin films as well as to the difference in the absorbances of double stacks: the Glass/IZrO bott/IZrO top stack absorbed more than the Glass/IZrO bott/AZO top double stack.

As far as the E_gap_ is concerned, in the asymmetric stack we found a value of 3.38 eV related to the AZO band gap, while in the symmetric-stack Glass/IZrO bott/IZrO top we found a value of 3.58 associated with the In_2_O_3_ band gap. These E_gap_ values calculated for AZO and IZrO were in good agreement with the values found in the literature [24,25,33]. Moreover, the E_gap_ calculated by the Tauc plot was affected by an error of 0.05 eV; the energy gap values of all stacks are listed in Table 1.

**Figure 4:**
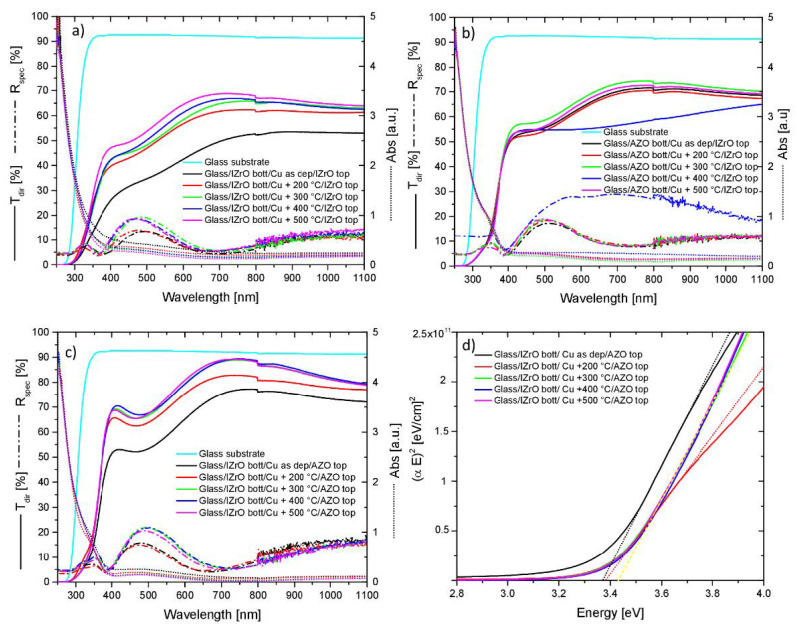
Transmittance, reflectance, and absorbance curves of dry PCS as a function of the annealing temperature with all combinations of AZO and IZrO layers as the top and bottom TCO: (**a**) IZrO_bott_/Cu/IZrO_top_; (**b**) AZO_bott_/Cu/IZrO_top_; (**c**) IZrO_bott_/Cu/AZO_top_. The transmittance of the bare glass substrate is reported as a reference. (**d**) Tauc plots and calculated linear fit of the samples reported in (**c**).

Regarding the dry PCS, we observed that in asymmetric structures (Figure 4b,c), independently of the annealing temperature, a threshold around 364 nm was recognized with a concurrently increasing absorbance from 364 nm to a lower wavelength in the UV region; while for the symmetric PCS composed of only IZrO layers (Figure 4a), once again, independently of the annealing temperature, we observed a threshold around 344 nm with a concurrently increasing absorbance from that value to a lower wavelength. We noted that for both the asymmetric and symmetric PCSs, when the Cu as-deposited thin film was deposited between TCOs, the threshold moved to a higher wavelength with respect to the thresholds previously mentioned. The thresholds were connected to the direct optical band gaps of the AZO layer and IZrO layer: sunlight photons with an energy higher than the energy band gap were absorbed by electrons in the valence band with their promotion to the conduction band. The values for the E_gap_ in the PCSs strongly depended on the type of top and bottom TCOs employed: the TCO with the lowest E_gap_ imposed the E_gap_ to the entire PCS.

In the 250–1100 nm wavelength range, we observed that symmetric and asymmetric PCSs showed the same behavior in the transmittance with respect to double stack but with higher values as a function of the annealing temperature and the evolution of Cu nanostructures embedded in the TCOs. On the other hand, the reflectance curves exhibited a complementary behavior with respect to transmittance, showing a maximum where the transmittance values were low and vice versa. In addition, in the visible–near-infrared range, the reflectance values for all structures were below 22%.

The best transmittance values were obtained for each type of symmetric and asymmetric dry PCS by Glass/IZrO_bott_/Cu + 500 °C/IZrO_top_, Glass/AZO_bott_/Cu + 300 °C/IZrO_top_, and Glass/IZrO_bott_/Cu + 400 °C/AZO_top_ with maximum values of 68.9% at 714 nm, 74.3% at 762 nm, and 89.4% at 754 nm, respectively. Among them, the best performance according to the mean values of the transmittance calculated in the visible range (400–800 nm) and visible–NIR range (400–1100 nm) belonged to Glass/IZrO_bott_/Cu + 400 °C/AZO_top_ with values of 78.7% and 80.8%, respectively; while Glass/IZrO_bott_/Cu + 500 °C/IZrO_top_ and Glass/AZO_bott_/Cu + 300 °C/IZrO_top_ showed values in that range of 61.2–63.0% and 66.0–68.5%, respectively.

For the best dry PCS (Glass/IZrO_bott_/Cu + 400 °C/AZO_top_), an analysis of the absorbance, transmittance, and reflectance curves allowed us to highlight that higher values of transmittance were obtained thanks to a very low absorbance even though the reflectance values were among the higher ones (21.9%) in the visible range. The Cu nanostructures embedded in TCOs contributed to an increase in the transmittance at the expense of the reflectance and absorbance.

Moreover, for the best dry structures (Glass/IZrO_bott_/Cu + different annealing temperatures/AZO_top_), we showed that the Tauc plots and calculated linear fit of the samples (Figure 4d) used to evaluate the Egap had band-gap values in the range of 3.37 to 3.43 eV. If we considered the Egap ± uncertainty range measured, then due to the overlap among values, the measurements were consistent. This meant that all these structures showed the same E_gap_ (for AZO). The E_gap_ values for all remaining structures are listed in Table 1.

**Figure 5:**
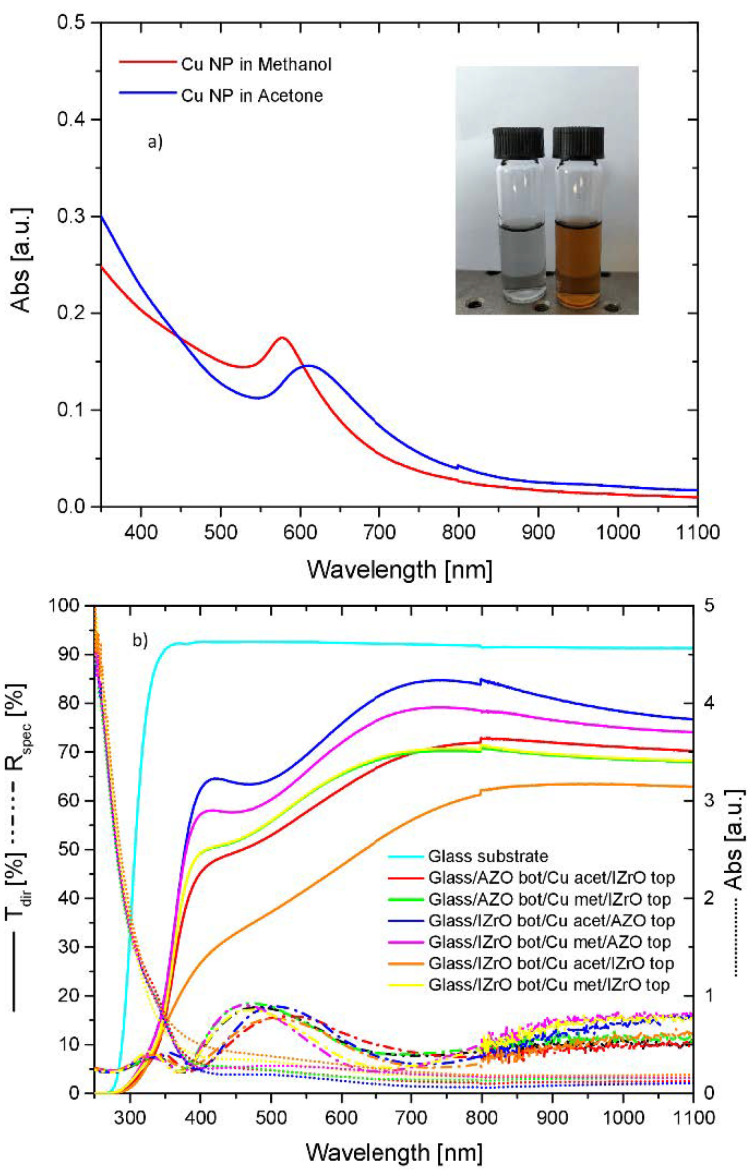
(**a**) Absorbance spectra of colloid solutions of metallic copper nanoparticles prepared in acetone (on the right side of the photo in the caption) and methanol (on the left side of the photo in the caption). (**b**) Transmittance, reflectance, and absorbance curves of wet systems as a function of the solvent employed to produce Cu nanoparticles and for all combination of TCOs as the top and bottom layer. The transmittance of the bare glass substrate is also reported for reference.

Figure 5a shows the absorbance spectra of colloidal copper solutions prepared in acetone and methanol at a laser ablation fluence of 5 J cm^−2^ per pulse and for an ablation time of 8 min.

Our aim was to produce almost-pure Cu metallic nanoparticles; therefore, water was avoided as a solvent due to its high oxidation power [18], while acetone and methanol were employed due to their characteristics of low toxicity, environmental safety, low cost, and a low oxidation power resulting from their low/moderately hygroscopicity [34].

The spectra as a function of the solvent employed exhibited a distinct SPR in the visible range with a copper interband transition (IBT) in the UV short-wavelength region. Specifically, the positions of the SPR band peaks were at 610 nm for acetone and 578 nm for methanol. The Cu colloidal solution obtained in acetone showed a red-shifted SPR peak compared to methanol along with differences in shape and intensity. The SPR peak in acetone was weak and broad while in methanol it was slightly more intense and narrow. A check of the Cu target mass before and after the process of laser ablation detected more Cu dissolved in acetone (25 10^−5^ g) compared to that in methanol (16 10^−5^g); these differences mainly arose from the difference in particle sizes, which were larger in methanol than in acetone.

After the preparation of the Cu colloidal solutions in acetone and methanol, the nanoparticles were embedded between the TCO layers (see wet PCS synthesis). The transmittance, reflectance, and absorbance spectra of all wet PCSs as a function of the solvent employed (to create Cu nanoparticles) and for all combinations of AZO and IZrO as the top and bottom layer are shown in Figure 5b. Moreover, the transmittance of the glass substrate is reported and, as in all cases, the optical data included the glass substrate’s contribution.

As far as the transmittance is concerned, all wet asymmetric PCSs once again showed, independent of the colloidal solutions (and metallic copper nanoparticles) employed, a threshold around 364 nm and, simultaneously, a considerable increase in the absorbance in the UV region from 364 nm. For the symmetric PCSs, independent of the colloidal solutions, we observed a threshold around 344 nm and, simultaneously, an increase in the absorbance from 344 nm to a lower wavelength. As stated previously, these thresholds were related to the direct optical band gaps of the AZO layers and IZrO layer, respectively.

Once again, in the visible–NIR range (400–1100 nm), we observed that the symmetric and asymmetric PCSs showed the same behavior in the transmittance with respect to double stacks but with higher values as a function of the type of Cu nanoparticles (and solvent used). On the other hand, the reflectance curves exhibited a complementary behavior with respect to the transmittance behavior, showing a maximum when the transmittance values were low and vice versa. However, in this case the reflectance values for all structures were below 20%.

The best transmittance values among all the symmetric and asymmetric wet PCSs were achieved by Glass/IZrO_bott_/Cu Np in acetone/AZO_top_ followed by Glass/IZrO_bott_/Cu Np in methanol/AZO_top_, with maximum values of 84.7% at 737 nm and 79.2% at 745 nm, respectively. The best performance according to the mean values of the transmittance calculated in the visible range and visible–NIR range belonged to Glass/IZrO_bott_/Cu Np in acetone/AZO_top_, with values of 74.7% and 76.9%, respectively. 

For the best wet PCS (Glass/IZrO_bott_/Cu Np in acetone/AZO_top_), an analysis of the absorbance, transmittance, and reflectance curves allowed us to highlight that higher values for transmittance were obtained thanks to very low absorbance and reflectance values, which were lower than 20% in all wavelength ranges analyzed. In addition, in this case we observed, from 364 nm to a higher wavelength, that the increase in the transmittance in the wet system was related to a decrease in the reflectance and absorption compared to the reference sample (Glass/IZrO_bott_/AZO_top_).

### 2.3. Electrical and Optical (E_gap_) Properties

The sheet resistances of the wet and dry PCSs along with those of the AZO and IZrO thin films, AZO/IZrO, IZrO/AZO, and IZrO/IZrO double stack as references, were measured at room temperature and are reported in Table 1 and Table 2.

The AZO thin film layer deposited at room temperature was characterized by a very high value of R_sh_. This value depended on the thickness, TCO and Al doping nature, and deposition-process parameters such as room temperature deposition [23,24,25]. Conversely, the IZrO thin film deposited at room temperature showed a very low value of about 200–300 Ω/sq. Briefly, the IZrO film was grown using magnetron sputtering with zirconium defect levels sufficiently high in energy and above the conduction band minimum of In_2_O_3_ so that the transition metal dopant could be fully ionized at room temperature and act as a shallow donor along with high values of the electron concentration (n_e_) and electron mobility (µ_e_) [35,36,37,38,39].

The R_sh_ of the TCO double stacks in all configurations could be modeled while considering the bottom TCO and top TCO layers as two resistors connected in parallel; in this case, we considered only the part between the brackets in Equation (3). Therefore, if we had two TCOs with a high difference in sheet resistance values, the R_sh_ of the double stack was dominated by the TCO with the lowest sheet resistance, while it was about R_sh_/2 if TCOs had the same (or a very close) sheet resistance; e.g., the IZrO/IZrO stack.

When Cu in both the dry and wet configurations was deposited between the TCO layers, the R_sh_ value of this three-layer stack could be seen as three resistors connected in parallel (Equation (3) with all elements). If we take into account that:

- In all the dry PCSs, the Cu film as deposited was a noncontinuous film or coalesced gradually toward larger particles as a function of thermal annealing;
- In all the wet PCSs, Cu NPs with various sizes were randomly and uniformly distributed on bottom TCOs;
- Cu in all its forms in these structures showed a very high sheet resistance >106 Ω/sq, the sheet resistance of all wet and dry PCSs only depended on the two TCO layers due to a negligible value for the Cu sheet resistance:

1/R_system_ = (1/R_sh, bott TCO_ + 1/R_sh, top TCO_) + 1/R_sh,Cu_ (3)

If we considered the first configuration (Glass/AZO bottom/Cu (as deposited or + thermal annealing)/IZrO top), as expected, after a thermal annealing we observed an improvement in the AZO bottom R_sh_ of up to three orders of magnitude until about 2 × 10^3^ Ω/sq at 400 °C [40]. Beyond 400 °C, no significant improvement was observed. In the meantime, the AZO bottom layer improved its Rsh as a function of temperature while the Cu coalesced gradually toward a non continuous film or larger unconnected nanoparticles.

Finally, with the room temperature deposition of IZrO top layer, the structure could be seen as three resistors connected in parallel. Here, the entire R_sh_ was controlled by the IZrO top layer due to its lowest sheet resistance as compared to all types of annealed AZO bottom layers and all forms of Cu. In fact, in all of these types of structures, we observed very similar sheet resistance values to that of the IZrO thin film.

In the second configuration (Glass/IZrO bottom/Cu (as deposited or + thermal annealing)/AZO top), we observed an improvement in the R_sh_ value up to 200 °C; then, for higher annealing temperatures, a gradual worsening up to ~ 4.4 × 10^3^ Ω/sq. If we considered the sheet resistance of the AZO top layer deposited at room temperature and how the layers of structure influenced the R_sh_ of all of the systems, we could presume that IZrO improved the electrical properties up to 200 °C, then it degraded. Here, once again, the IZrO layer controlled the R_sh_ of the PCS.

In the third configuration (Glass/IZrO bottom/Cu (as deposited or + thermal annealing)/IZrO top), when considering the behavior of the IZrO sheet resistance as a function of temperature and the condition that at room temperature both IZrO layers (top and bottom) had the same sheet resistance, we observed a smaller gradual worsening up to 521 Ω/sq at 500 °C. In this case, the upper layer of IZrO deposited at room temperature compensated for the worsening of the IZrO bottom layer.

On the other hand, for the wet PCSs, with the previous considerations for TCOs layers deposited at room temperature along with the bottom TCO layer undergoing an irrelevant thermal treatment on a hot plate (100 °C, 30 min), we observed, as expected, that in the asymmetric wet PCS, the R_sh_ was controlled by the IZrO layer, while in the symmetric PCS with both IZrO layers, the R_sh_ was about R_sh, IZrO_/2.

### 2.4. Comparing the best PCSs

**Figure 6:**
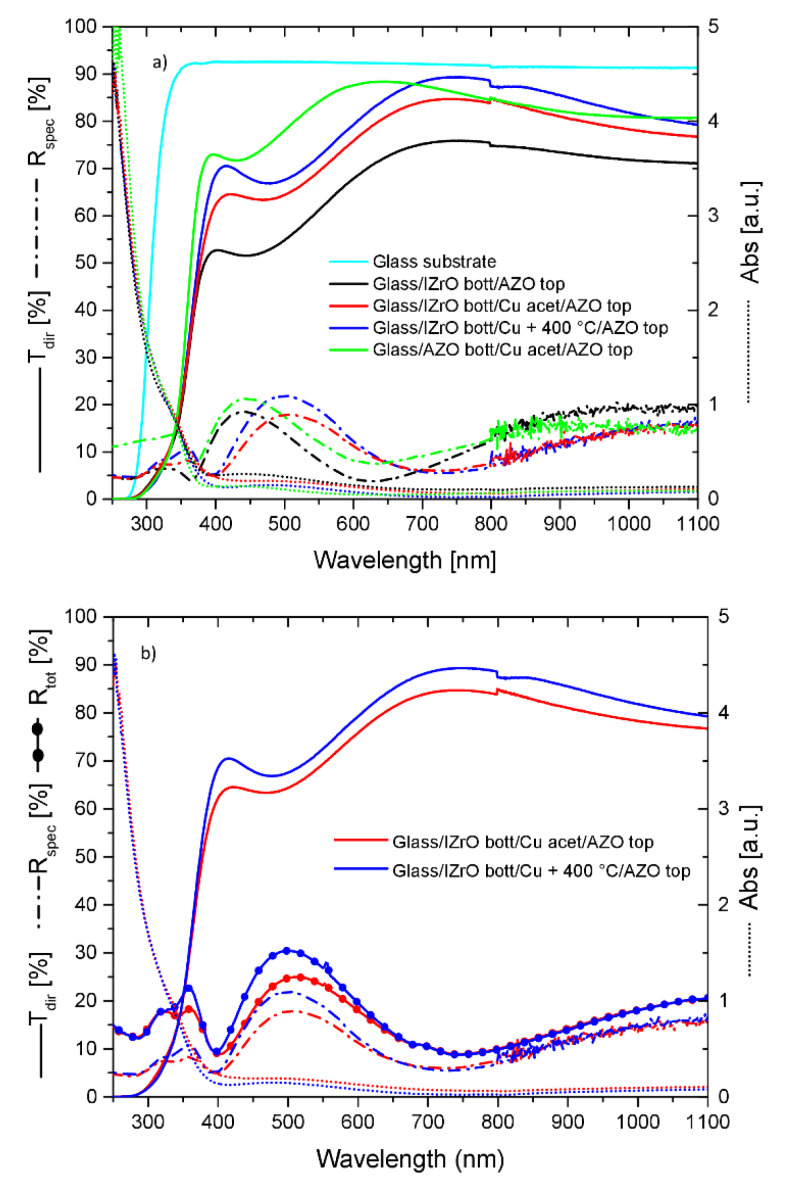
(**a**) Comparison of direct transmission, specular reflectance, and absorbance of the best selected structures. Glass substrate and Glass/IZrO_bott_/AZO_top_ are reported as reference points. (**b**) Comparison of direct transmission, total reflectance, and absorbance of wet (Glass/IZrO/Cu Acet/AZO) and dry (Glass/IZrO/ Cu + 400 °C/AZO) structures.

Figure 6a,b and Table 3 show a comparison among the PCSs with the best optical and electrical properties produced in this work and in our previous work [40] for PCSs constructed using the wet procedure with AZO as the top and bottom layers.

Beginning with the electrical properties, it can be seen that the best performance was achieved by the structure with the IZrO layer incorporated, and in particular without thermal annealing. Between the best and the worst performances, there was a difference of about four orders of magnitude, with a best value of 253 Ω/sq.

On the other hand, from the optical point of view, no remarkable differences were observed in the E_gap_ caused by the presence of an AZO layer in all structures and therefore with values very close to each other and inside the affected error. The most remarkable modifications concerned the T, R, and abs values. In particular, as shown in Figure 6a and in the transmittance mean values in the visible and visible–NIR ranges reported in Table 3, the best performances were achieved by Glass/AZO_bott_/Cu Np acetone/AZO_top_ followed by Glass/IZrO_bott_/Cu + 400 °C/AZ and Glass/IZrO_bott_/Cu Np in acetone/AZO_top_.

As can be seen, these differences in transmittance arose from the different manners of absorption and the maximum (and minimum) position of reflectance. Between the best and the third-best in the visible and visible–NIR range, we found a difference of 8.1% and 5.5%, respectively, with the lowest absorption by Glass/AZO_bott_/Cu Np acetone/AZO_top_ and the lowest reflectance values (ranging from 5 to 18%) by Glass/IZrO_bott_/Cu Np in acetone/AZO_top_.

When comparing all the electrical and optical properties, although Glass/AZO_bott_/Cu NPs acetone/AZO_top_ showed the best optical performance, at the same time it also showed the worst electrical performance; therefore, it could not be selected as a PCS.

Between the wet and dry asymmetric PCSs, in order to make the best choice, we studied the total reflectance (specular + diffused) of light by these structures (Figure 6b). If we consider the components of the total transmittance, total reflectance, and the absorbance, we get:

(4) $$100\%=T_{tot}+R_{tot}+abs=T_{dir}+T_{diff}+R_{spec}+R_{diff}+abs$$

We observed that because both structures showed a very close absorption, with the higher total reflectance and T_dir_ belonging to the dry PCS, this implied that the wet PCSs had a higher T_diff_ part of the light. In particular, we calculated diffused light values ranging in the best conditions from 5 to 28% in the entire visible range. The higher values for T_diff_ were from 500 nm to lower wavelengths. In conclusion, the PCS with the best electrical and optical properties was Glass/IZrO_bott_/Cu Np in acetone/AZO_top_ because it showed the lowest sheet resistance, a good direct transmission, and the highest diffused transmission in the visible and NIR wavelength ranges.

## 3. Materials and Methods

AZO films were deposited onto a Corning glass 2947 substrate by means of RF magnetron sputtering using the following parameters: ceramic AZO target (2 wt% Al_2_O_3_, 98 wt% ZnO), power density of 2.16 W/cm^2^, 7 cm target–substrate distance, room temperature deposition, and argon working pressure of 5.7 × 10^−3^ mbar.

IZrO films were deposited onto a Corning glass 2947 substrate via an RF and DC magnetron cosputtering technique using a Zr (99.99% purity) and In_2_O_3_ target (2-inch diameter, 99.99% purity) supplied by Moorfield Nanotechnology. The cosputtering deposition of all films was performed by applying a 45 W sputtering power for In_2_O_3_ (in RF mode) and 57 W for Zr in (DC mode) at room temperature in an argon atmosphere with a working pressure of 1.6 × 10^−2^ mbar and a deposition time of 60 min. The sputtering system was used in a sputter-up configuration with a rotating sample holder.

In the dry configuration, Cu nanoparticles were fabricated by dewetting of a Cu thin film sputtered on both AZO or IZrO films. The Cu thin film was deposited by means of sputtering using an Emitech K550X sputter coater in conditions of 50 mA for 35 s. The thermal solid dewetting of the Cu thin films was carried out in a tubular oven made by Carbolite Gero in a saturated N_2_ atmosphere to avoid spontaneous oxidation at temperatures ranging from 200 to 500 °C for 60 min. Moreover, we took the precaution of waiting for a few minutes between the time (at room temperature) of depositions (Cu thin film and top TCO) and the thermal annealing. In this short time, no significant oxidation occurred [41].

In the wet configuration, the Cu nanostructures were produced via pulsed laser ablation from a Cu metal target (thickness of 1.0 mm, purity of 99.99%) immersed in methanol and acetone. The laser ablation was performed with a Nd:YAG laser (Spectra Physics, Santa Clara, CA, USA) using the following parameters: λ = 1064 nm,10 ns pulse, 5 J/cm^2^ fluence, 10 Hz frequency, and an ablation time of 8 min. A 2 mm laser spot was focused with a 100 mm focal length lens on the Cu target at the bottom of a Teflon vessel filled with 8 mL of methanol or acetone. Cu nanostructures from the colloidal solutions were transferred onto the bottom TCOs surface using a drop-casting method.

Rutherford backscattering spectrometry (RBS) was used to determine the stoichiometry and the areal density of: zinc (Zn), aluminum (Al), and oxygen (O) in the AZO layer; indium (In), oxygen (O), and zirconium (Zr) in the IZrO layer; and copper (Cu) in the Cu thin-film layer. A standard RBS analysis was performed with a 2.0 MeV He^+^ ion beam at normal incidence with the backscattered ions detected at an angle of 165°. SimNra software [42] was used to simulate the RBS spectra for the quantitative analysis. Once the doses of the chemical elements in the films were quantified, we converted them into thickness by using the atomic density of the material: AZO (4.22 × 10^22^ at./cm^3^), Cu (8.48 × 10^22^ at./cm^3^), and IZrO (7.69 × 10^22^ at/cm^3^). The thickness of AZO, IZrO, and Cu thin film was set at 85, 65, and 1.3 nm, respectively.

The IZrO and AZO surfaces along with the Cu nanoparticles’ morphology, were investigated by means of a scanning electron microscope (field-emission Gemini 152 Carl Zeiss Supra 25). The analyses were carried out by using the in-lens detector with an aperture size of 30 μm, a working distance of 3 mm, and an acceleration voltage of 3 kV. Moreover, the use of SEM associated with an EDAX-EDX detector was employed for an energy-dispersive X-ray analysis in order to identify the chemical composition (and distribution of elements) in our structure via the collection of characteristic X-rays emitted by the sample and induced by the primary electron beam (at 15 KeV). In particular, SEM images were processed to determine the average diameter of the Cu nanoparticles while assuming a spherical shape on a statistical population of at least 100 nanoparticles.

A four-point collinear probe method employing a Keithley 4200 semiconductor characterization system was used at room temperature to measure the sheet resistance (R_sh_) of all systems.

The direct transmittance (T_dir_), specular reflectance (R_spec_), and absorbance (Abs) spectra of samples in the 250–1100 nm wavelength range were measured by using a Varian Cary 500 double-beam scanning UV–vis–NIR spectrophotometer. In particular, the reflectance spectra were measured in specular geometry at 20° using a silicon-calibrated sample as reference, while the direct transmittance spectra were normalized to a 100% baseline obtained using an empty sample holder. Lastly, the total reflectance (R_tot_) was measured using a Perkin Elmer Lambda 40 UV–vis spectrometer equipped with an integrating sphere.

### Dry and Wet PCS Preparation

**Figure 7:**
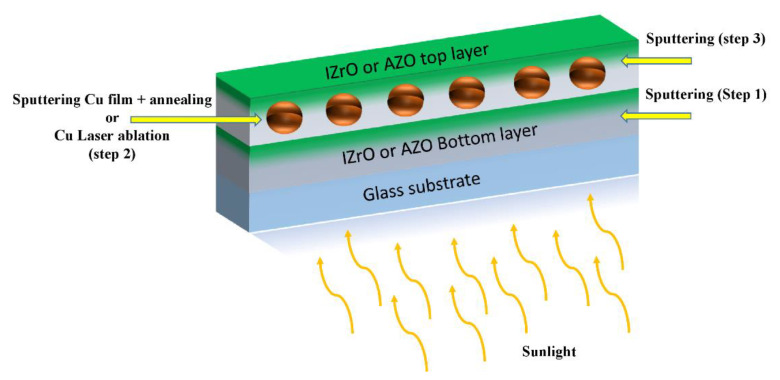
Steps sequence with the related processes that took place to fabricate the dry or wet PCSs.

PCSs in both dry and wet configurations were produced in three sequential steps (Figure 7).

The first step was the deposition of the bottom TCO (AZO or IZrO) layer on the glass substrate, then (second) the deposition of the Cu nanoparticles on the bottom TCO layer, and finally (third) the deposition of the top TCO (AZO or IZrO) layer in order to cover and protect the Cu nanoparticles. The TCO_bott_/Cu nanoparticle/TCO_top_ structures were synthesized using all combinations of AZO and IZrO as the top and bottom layers.

As a function of the method used to produce the Cu nanostructures, the second step was:

- In the dry configuration, after the deposition of the Cu thin film on the bottom TCO (AZO or IZrO), the Cu nanoparticles were produced by a thermal annealing of the glass substrate/bottom TCO/Cu thin-film stack. This led to an energetically favored set of Cu nanoparticles at temperatures below the melting point. Lastly, the previous Cu nanoparticles were covered with a top TCO (AZO or IZrO) layer.
- In the wet configuration, in the case of laser ablation in a solvent, after using an ultrasonic dispersion process on the produced colloidal solutions in order to avoid agglomeration of nanoparticles, a fraction measuring 100 µL was poured onto the bottom TCO layer. The solvent was evaporated by means of a hot plate at 100 °C for 30 min. This weak thermal process involved the glass substrate/bottom TCO/Cu nanoparticles + solvent stack. After the evaporation of the solvent, the top TCO layer was deposited.

## 4. Conclusions and Future Perspectives

In conclusion, Cu nanoparticles were produced by means of dry SSD of a 1.3 nm Cu layer and wet LAL of a Cu target using a 1064 nm nanosecond-pulsed laser in acetone and methanol. The realized Cu nanostructures were then embedded in TCOs such as an AZO layer or IZrO layer. The TCO_bott_/Cu nanoparticle/TCO_top_ structures were synthesized using all combinations of AZO and IZrO as the top and bottom layers. The goal of this research was to achieve a plasmonic and conductive structure (PCS) for photovoltaic applications via a comparison of the involved methods and all created structures.

The differences found between the wet and dry processes were in the shapes and sizes of the Cu nanoparticles: SSD produced faceted or quasispherical nanoparticles with an average size of less than 150 nm, while LAL produced spherical nanoparticles that were smaller than 50 nm in acetone and smaller than 250 nm in methanol. The dry and wet PCSs as a function of the TCOs employed and temperature of annealing could achieve a sheet resistance of 86 Ω/sq in the best conditions and ~250 Ω/sq at room temperature for the wet and dry PCSs. The optical properties such as the energy band gap, transmittance, absorbance, and specular or diffused reflectance investigated in the 250–1100 nm UV–vis–NIR range showed a dependence on the sequence of the top and bottom TCOs and type (wet or dry) of Cu nanoparticles embedded. The values for the E_gap_ in the PCSs strongly depended on the type of top and bottom TCOs employed: the TCO with the lowest Egap imposed the E_gap_ to the entire PCS. The best dry PCS exhibited a maximum transmittance of 89.4% at 754 nm with a mean value of transmittance in the visible range of 78.7% and in the visible–NIR range of 80.8%; while the best wet PCS in acetone showed 84.7% at 737 nm, 74.7% in the visible range, and 76.9% in the visible–NIR range. Moreover, the best wet PCS showed a higher diffused transmittance of between 10 and 20% in the visible range compared to the best dry PCS. If we considered the dimensions of the Cu nanoparticles in both the wet and dry configurations, the optical behaviors, especially for the diffused part of the transmitted light, were in agreement with the literature [43,44]: in small NPs (d < 10 nm), the absorption dominated while the scattering increases strongly with the dimensions up to a saturation of d > 100 nm.

The present work can be considered as a second step toward the optimization of Cu nanoparticles and transparent conductive oxides in order to design and engineer the best PCS for improving solar cell devices. Therefore, based on this research and our previous study, our next step will be the fabrication of Si-based solar cell prototypes by growing a Si-based active layer on a PCS and then testing of the performance of the prototypes.

## Figures and Tables

**Table 1:** Sheet resistances and Egap of AZO and IZrO thin film layers; AZO/IZrO, IZrO/AZO, and IZrO/IZrO double stacks; and dry systems with all combinations of AZO and IZrO layers as the top and bottom TCO.

Samples	R_sh_ (Ω/Sq)	E_gap_
AZO thin film	1 × 10^6^	3.41
IZrO thin film	243	3.58
AZO bott/IZrO top	235	3.38
IZrO bott/AZO top	236	3.38
IZrO bott/IZrO top	178	3.58
Samples	R_sh_ (Ω/Sq)	E_gap_
AZO_bott_/Cu as dep/IZrO_top_	268	3.36
AZO_bott_/Cu + 200 °C/IZrO_top_	279	3.37
AZO_bott_/Cu + 300 °C/IZrO_top_	287	3.38
AZO_bott_/Cu + 400 °C/IZrO_top_	274	3.38
AZO_bott_/Cu + 500 °C/IZrO_top_	277	3.40
Samples	R_sh_ (Ω/Sq)	E_gap_
IZrO_bott_/Cu as dep/AZO_top_	265	3.37
IZrO_bott_/Cu + 200 °C/AZO_top_	83	3.38
IZrO_bott_/Cu + 300 °C/AZO_top_	1019	3.43
IZrO_bott_/Cu + 400 °C/AZO_top_	2093	3.43
IZrO_bott_/Cu + 500 °C/AZO_top_	4385	3.43
Samples	R_sh_ (Ω/Sq)	E_gap_
IZrO_bott_/Cu as dep/IZrO_top_	153	3.51
IZrO_bott_/Cu + 200 °C/IZrO_top_	87	3.62
IZrO_bott_/Cu + 300 °C/IZrO_top_	289	3.62
IZrO_bott_/Cu + 400 °C/IZrO_top_	322	3.61
IZrO_bott_/Cu + 500 °C/IZrO_top_	521	3.64

**Table 2:** Wet systems with all combination of AZO and IZrO layers as the top and bottom TCO.

Samples	R_sh_ (Ω/Sq)	E_gap_
AZO_bott_/Cu acet/IZrO_top_	304	3.38
AZO_bott_/Cu met/IZrO_top_	263	3.37
IZrO_bott_/Cu acet/AZO_top_	253	3.39
IZrO_bott_/Cu met/AZO_top_	215	3.38
IZrO_bott_/Cu acet/IZrO_top_	124	3.58
IZrO_bott_/Cu met/IZrO_top_	132	3.57

**Table 3:** Comparison of sheet resistance, energy band gap, mean value T in visible (400–800 nm) and visible–NIR (400–1100 nm) ranges of the best selected structures. Glass/IZrO_bott_/AZO_top_ is reported as a reference point. The data for the AZO_bott_/Cu acetone/AZO_top_ sample are from our previous work [40].

Samples	R_sh_ (Ω/Sq)	E_gap_	<T>_Vis_ (%)	<T>_Vis-NIR_ (%)
IZrO_bott_/AZO_top_	236	3.38	65.4	68.5
IZrO_bott_/Cu Acet/AZO_top_	253	3.38	74.7	76.9
IZrO_bott_/Cu + 400 °C/AZO_top_	2093	3.43	78.7	80.8
AZO_bott_/Cu acet/AZO_top_ [40]	2.5 × 10^6^	3.44	82.8	82.4

## Data Availability

Not applicable.

## Supplementary Materials

The following are available online at https://www.mdpi.com/article/10.3390/ijms231911886/s1.

## References

[1] Comunication from the Commission to the European Parliament, the Council, the European Economic and Social Committee and the Committee of the Regions. EU Solar Energy Strategy–Brussels. https://eur-lex.europa.eu/legal-content/EN/TXT/?uri=COM%3A2022%3A221%3AFIN.

[2] Kumar N.M., Chopra S.S., de Oliveira A.K., Ahmed H., Vaezi S., Madukanya U.E., Castañón J.M., Gorjian S., Shukla A. (2020). Solar PV module technologies. Photovoltaic Solar Energy Conversion.

[3] Bailey W.L., Coleman M.G., Harris C.B., Lesk I.A. (1979). Texture etching of silicon: Method. U.S. Patent.

[4] Campbell P., Green M.A. (1987). Light trapping properties of pyramidally textured surfaces. J. Appl. Phys..

[5] Campbell P., Green M.A. (2001). High performance light trapping textures for monocrystalline silicon solar cells. Sol. Energy Mater. Sol. Cells.

[6] Enrichi F., Quandt A., Righini G.C. (2018). Plasmonic enhanced solar cells: Summary of possible strategies and recent results. Renew. Sustain. Energy Rev..

[7] De Aberasturi D.J., Serrano-Montes A.B., Liz-Marzán L.M. (2015). Modern Applications of Plasmonic Nanoparticles: From Energy to Health. Adv. Optical Mater..

[8] Li Y.F., Kou Z.L., Jing F., Hong-Bo S. (2020). Plasmon-enhanced organic and perovskite solar cells with metal nanoparticles. Nanophotonics.

[9] De Souza M.L., Corio P., Brolo A.G. (2012). Cu nanoparticles enable plasmonic-improved silicon photovoltaic devices. Phys. Chem. Chem. Phys..

[10] Shen P., Liu Y., Long Y., Shen L., Kang B. (2016). High-Performance Polymer Solar Cells Enabled by Copper Nanoparticles-Induced Plasmon Resonance Enhancement. J. Phys. Chem. C.

[11] Parveen F., Sannakki B., Mandke M.V., Pathan H.M. (2016). Copper nanoparticles: Synthesis methods and its light harvesting performance. Sol. Energy Mater. Sol. Cells.

[12] Zheng P., Tang H., Liu B., Kasani S., Huang L., Wu N. (2019). Origin of strong and narrow localized surface plasmon resonance of copper nanocubes. Nano Res..

[13] Kitcometals. http://www.kitcometals.com/charts/.

[14] NASDAQ. https://www.nasdaq.com/market-activity.

[15] Chan G.H., Zhao J., Hicks E.M., Schatz G.C., Van Duyne R.P. (2007). Plasmonic Properties of Copper Nanoparticles Fabricated by Nanosphere Lithography. Nano Lett..

[16] Sugawa K., Tamura T., Tahara H., Yamaguchi D., Akiyama T., Otsuki J., Kusaka Y., Fukuda N., Ushijima H. (2013). Metal-Enhanced Fluorescence Platforms Based on Plasmonic Ordered Copper Arrays: Wavelength Dependence of Quenching and Enhancement Effects. ACS Nano.

[17] Baruah P.K., Sharma A.K., Khare A. (2019). Role of confining liquids on the properties of Cu@Cu_2_O nanoparticles synthesized by pulsed laser ablation and a correlative ablation study of the target surface. RSC Adv..

[18] Liu P., Wang H., Li X., Muchen R., Zeng H. (2015). Localized surface plasmon resonance of Cu nanoparticles by laser ablation in liquid media. RSC Adv..

[19] Rawat R., Tiwari A., Arun N., Rao S.V.S.N., Pathak A.P., Tripathi A. (2019). Solvents effect on the morphology and stability of cu/cuo nanoparticles synthesized at high fluence laser ablation. ChemistrySelect.

[20] Crane C.C., Wang F., Li J., Tao J., Zhu Y., Chen J. (2017). Synthesis of Copper–Silica Core–Shell Nanostructures with Sharp and Stable Localized Surface Plasmon Resonance. J. Phys. Chem. C.

[21] Gawande M.B., Goswami A., Felpin F., Asefa T., Huang X., Silva R., Zou X., Zboril R., Varma R.S. (2016). Cu and Cu-Based Nanoparticles: Synthesis and Applications in Catalysis. Chem. Rev..

[22] Tilaki R.M., Iraji Zad A., Mahdavi S.M. (2007). Size, composition and optical properties of copper nanoparticles prepared by laser ablation in liquids. Appl. Phys. A.

[23] Ellmer K. (2012). Past achievements and future challenges in the development of optically transparent electrodes. Nat. Photonics.

[24] Boscarino S., Torrisi G., Crupi I., Ruffino F., Terrasi A. (2017). Ion irradiation of AZO thin films for flexible electronics. Nucl. Instrum. Methods Phys. Res. Sect. B Beam Interact. Mater. At..

[25] Ginley D.S., Hosono H., Paine D.C. (2010). Handbook of Transparent Conductors.

[26] Ruffino F., Grimaldi M.G. (2015). Controlled dewetting as fabrication and patterning strategy for metal nanostructures. Phys. Status Solidi A.

[27] Censabella M.V., Torrisi V., Boninelli S., Bongiorno C., Grimaldi M.G., Ruffino F. (2019). Laser ablation synthesis of mono- and bimetallic Pt and Pd nanoparticles and fabrication of Pt-Pd/Graphene nanocomposites. Appl. Surf. Sci..

[28] Ghorannevis Z., Akbarnejad E., Ghoranneviss M. (2015). Structural and morphological properties of ITO thin films grown by magnetron sputtering. J. Theor. Appl. Phys..

[29] Baladi A., Sarraf Mamoory R. (2010). Investigation of different liquid media and ablation times on pulsed laser ablation synthesis of aluminum nanoparticles. Appl. Surf. Sci..

[30] Naser H., Alghoul M.A., Hossain M.K., Asim N., Adbullah M.F., Ali M.S., Alzubi F.G., Amin N. (2019). The role of laser ablation technique parameters in synthesis of nanoparticles from different target types. J. Nanoparticle Res..

[31] Tauc J., Bagley B.G. (1977). Amorphous and Liquid Semiconductors.

[32] Mirabella S., Agosta R., Franzó G., Crupi I., Miritello M., Savio R.l., di Stefano M.A., di Marco S., Simone F., Terrasi A. (2009). Light absorption in silicon quantum dots embedded in silica. J. Appl. Phys..

[33] Dasari S.G., Nagaraju P., Yelsani V., Tirumala S., Reddy M.V.R. (2021). Nanostructured Indium Oxide Thin Films as a Room Temperature Toluene Sensor. ACS Omega.

[34] Tan B., Melius P., Ziegler P. (1982). A simple gas chromatographic method for the study of organ solvents: Moisture analysis, hygroscopicity, and evaporation. J. Chromatogr..

[35] Xu J., Liu J.B., Liu B.X., Li S.N., Wei S.H., Huang B. (2018). Design of n-Type Transparent Conducting Oxides: The Case of Transition Metal Doping in In_2_O_3_. Adv. Electron. Mater..

[36] Liang Y.C., Liang Y.C. (2009). Physical properties of low temperature sputtering-deposited zirconium-doped indium oxide films at various oxygen partial pressures. Appl. Phys. A.

[37] Rucavado E., Landucci F., Döbeli M., Jeangros Q., Boccard M., Hessler-Wyser A., Ballif C., Morales-Masis M. (2019). Zr-doped indium oxide electrodes: Annealing and thickness effects on microstructure and carrier transport. Phys. Rev. Mater..

[38] Aissa B., Zakaria Y., Shetty A.R., Samara A., Broussillou C. High Electron-Mobility of a Transparent and Conductive Zr-Doped In_2_O_3_Deposited by Reactive Magnetron Sputtering. Proceedings of the Conference Record of the IEEE Photovoltaic Specialists Conference.

[39] Morales-Masis M., Rucavado E., Monnard R., Barraud L., Holovsky J., Despeisse Y., Boccard M., Ballif C. (2018). Highly Conductive and Broadband Transparent Zr-Doped In_2_O_3_ as Front Electrode for Solar Cells. IEEE J. Photovolt..

[40] Boscarino S., Censabella M., Micali M., Russo M., Terrasi A., Grimaldi M.G., Ruffino F. (2022). Morphology, Electrical and Optical Properties of Cu Nanostructures Embedded in AZO: A Comparison between Dry and Wet Methods. Micromachines.

[41] Aromaa J., Kekkonen M., Mousapour M., Jokilaakso A., Lundström M. (2021). The Oxidation of Copper in Air at Temperatures up to 100 °C. Corros. Mater. Degrad..

[42] Ziegler J.F., Biersack J.P., Littmark U. (1984). The Stopping and Range of Ions in Solids, Stopping and Ranges of Ions in Matter.

[43] Morawiec S., Mendes M.J., Priolo F., Crupi I. (2019). Plasmonic nanostructures for light trapping in thin-film solar cells. Mater. Sci. Semicond. Process..

[44] Temple T.L., Bagnall D.M. (2013). Broadband scattering of the solar spectrum by spherical metal nanoparticles. Prog. Photovolt. Res. Appl..

