# Supplementary material for: Plasmonic and Conductive Structures of TCO Films with Embedded Cu Nanoparticles
Source: Int J Mol Sci. 2022 Oct 6;23(19):11886. doi: 10.3390/ijms231911886 (PMC9569451; doi:10.3390/ijms231911886)
Supplement: Supplementary file 1 [file ijms-23-11886-s001.zip › ijms-1923909-supplementary.pdf]

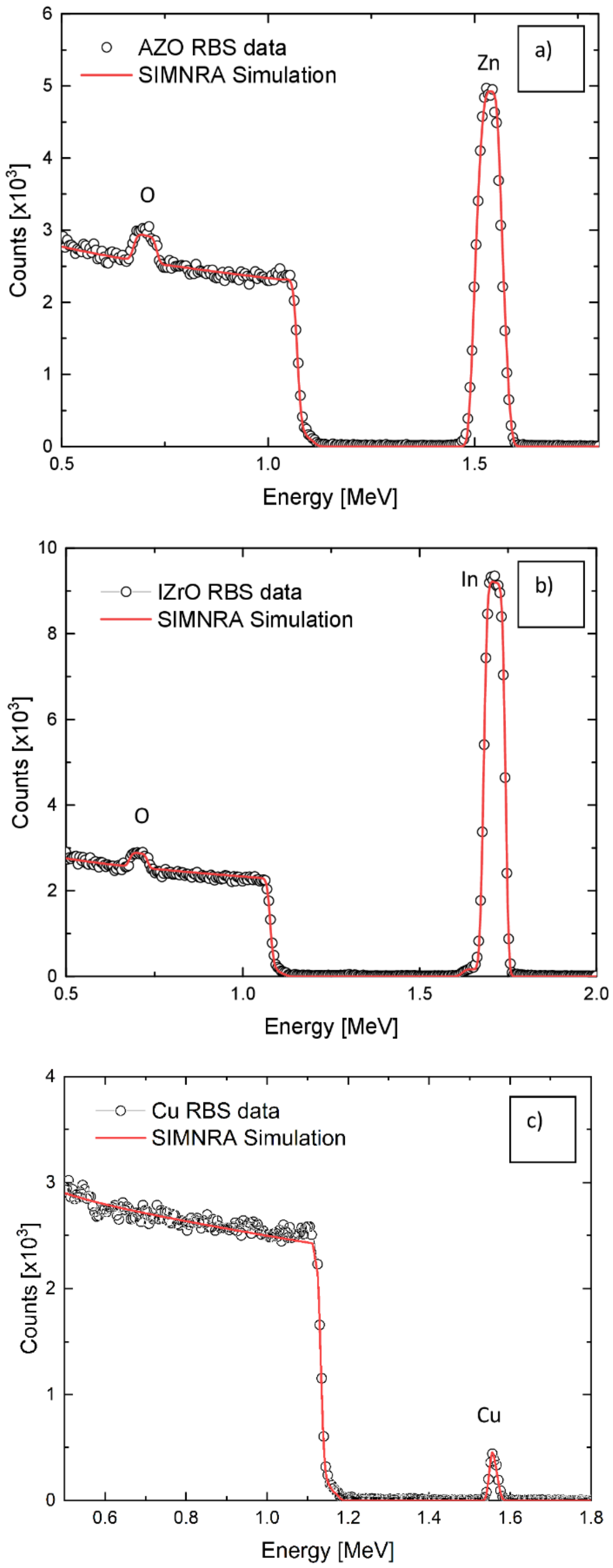

Figura S1: RBS extended experimental Spectra and Simulated curves with SimnNra software for a) AZO; b) IZrO; c) Cu thin films on Silicon substrate.
